# Performance of metagenomic next-generation sequencing for microbiological diagnosis of infectious uveitis

**DOI:** 10.1099/jmm.0.001879

**Published:** 2024-12-11

**Authors:** Zhen Cai, Xian Zhang, Yaqin Song, Yan Jiang, Ling Jiang, Tao Li, Xufang Sun

**Affiliations:** 1 Department of Ophthalmology, Tongji Hospital, Tongji Medical College, Huazhong University of Science and Technology, Wuhan, PR China; 2 Xiangyang Hospital affiliated to Hubei University of Chinese Medicine, Xiangyang, PR China

**Keywords:** mNGS, pathogenic spectrum, routine diagnostic tests, uveitis

## Abstract

**Introduction.** Diagnosis of uveitis is challenging due to the multitude of possible pathogenies. Identifying infectious and non-infectious uveitis is of great clinical significance. Recently, metagenomic next-generation sequencing (mNGS) was used to detect infectious and non-infectious uveitis, but its efficacy has not been widely evaluated.

**Hypothesis.** Compared with routine diagnostic tests (RDTs), mNGS is more effective in identifying infectious and non-infectious uveitis.

**Aim.** To describe the microbiological diagnostic performance of mNGS in detecting infectious and non-infectious uveitis.

**Methodology.** Patients with suspected infectious uveitis of uncertain pathogenesis were tested by mNGS and RDTs. Infectious and non-infectious uveitis were grouped according to the final diagnosis based on comprehensive analysis of the test results and the effect of therapy. The test results were used to assess the performance of mNGS in actual clinical practice.

**Results.** Fifty-eight cases were enrolled in this project, including 32 cases of infectious uveitis and 26 cases of non-infectious uveitis. The sensitivity of mNGS was 96.88%, which was much higher than that of RDTs. The detected pathogenic micro-organisms included bacteria, fungi, viruses, *Toxoplasma gondii* and *Bartonella*. Consequently, mNGS showed a high negative predictive value (NPV) of 94.74%, indicating that an mNGS negative should be a true negative result most of the time, but a low positive predictive value (PPV) of 79.49%.

**Conclusions.** mNGS showed extremely high sensitivity but low specificity, which increased the detection rate of infectious uveitis pathogens but might result in false positives. The excellent NPV suggested that the identification of non-infectious uveitis is of considerable clinical importance.

## Introduction

Uveitis is an important cause of ocular morbidity and blindness worldwide [1]. However, diagnosis is challenging due to the multitude of possible pathogenies. The existing paradigm for distinguishing between infections and non-infectious uveitis relies on the physician formulating a differential diagnosis based on the patient’s history and clinical presentation, followed by serial laboratory testing [2]. This traditional approach is particularly challenging for uveitis given overlapping clinical manifestations of infectious and non-infectious causes, a lack of diagnostic tests for rare pathogens, and the limited availability and volume of intraocular fluid samples owing to the requirement for invasive procedures. Thus, the exact causes of uveitis are difficult to identify in many patients. Failure to obtain a timely diagnosis in patients with uveitis contributes to poor patient outcomes, increased patient and family anxiety, and a high cost burden to the healthcare system [3].

Metagenomic next-generation sequencing (mNGS) is a culture-independent method for detecting infectious pathogens, particularly rare or novel pathogens, and it outperforms traditional methods in diagnosis, indicating its potential for use in early diagnosis [4]. Furthermore, when compared to traditional methods, mNGS takes only about 24 h, allowing for rapid pathogen identification in the case of complex diseases. This high-throughput technique has the potential to detect 17 500 pathogens in small amounts of clinical samples such as aqueous humour and vitreous humour, providing new evidence to guide anti-infection treatment. mNGS has been successfully used in a variety of infectious diseases, according to published case reports and clinical studies [4,8]. However, there is a lack of data on the use of mNGS in patients suspected of having infectious uveitis. This study was carried out at a large Chinese hospital to assess the performance of mNGS in patients suspected of having infectious uveitis.

We performed a prospective study involving hospitalized patients presenting with uveitis. We recently described the analytical sensitivity and specificity of the mNGS assay of intraocular fluid samples (aqueous humour and vitreous humour) for the identification of pathogens in uveitis patients confirmed by routine diagnostic tests (RDTs), including culture [9], PCR [10], antibodies [11] and other detection methods, such as T-spot [12] for suspected tuberculosis infection. In this regard, the current study was performed to expand mNGS testing to reach broader pathogens and samples while assessing its performance in real-life clinical practice.

## Methods

### Study design

#### Patients

This study was a single-centre, prospective case series in which patients were enrolled based on a particular presentation as uveitis, and then followed over time to assess outcomes. The project was approved by the Ethical Committee of Tongji Hospital, Tongji Tongji Medical College, Huazhong University of Science and Technology. Before entering the project, all participants were informed that they would need to go through a rigorous screening. Depending on the needs of the patient, some needed to undergo vitrectomy and vitreous humour was obtained at the same time, or aqueous humour was obtained by undergoing anterior puncture, and all the samples were used for mNGS testing and RDTs.

We enrolled 58 cases of suspected uveitis in which the pathogenesis was unclear. All participants signed a consent form for the programme and an informed consent form for anterior puncture or vitrectomy. The consent forms were approved by the Ethical Committee of Tongji Hospital, Tongji Tongji Medical College, Huazhong University of Science and Technology. We excluded uveitis patients with confirmed non-infectious diseases, such as HLA-B27-positive associated uveitis, Vogt–Koyanagi–Harada (VKH) syndrome and Behcet disease. Patients who were under 18 years of age or had already accepted specific antimicrobial therapy or refused the mNGS test were also excluded.

#### Tests

Before definitive treatment, all patients received both RDTs and mNGS tests. For patients suspected of being infected with virus according to the clinical manifestation, such as Cytomegalovirus (CMV), Herpes simplex virus (HSV), and Varicella-zoster virus (VZV), polymerase chain reaction (PCR) was the method of choice for rapid verification. Patients suspected of being infected with bacteria or fungi were tested by culture. For some suspected cases of infection with particular pathogenic micro-organisms, specific diagnostic tests were chosen, such as for *Toxoplasma gondii* (antibody in blood and aqueous humour), *Mycobacterium tuberculosis* complex [purified protein derivative (PPD) test, T-spot test and chest computed tomography (CT)] and *Bartonella henselae* (a history of cat scratches, PCR and antibody). At the same time, all patients received an mNGS test.

#### Samples

For uveitis patients who were scheduled for vitrectomy, we took vitreous humour as the test sample during surgery. Otherwise, we performed an anterior chamber puncture to achieve about 150 µl aqueous humour as the test sample before any therapy. All samples were refrigerated and transported to the laboratory. Once at the laboratory, the samples were frozen and stored in a −80 °C freezer. Tests were started within 24 h.

In some cases, we were not sure if a vitrectomy was to be performed, so we took aqueous humour as the sample. However, some of these cases were treated with vitrectomy, and thus vitreous humour was used for laboratory tests. For these cases, only the results of the aqueous humour sample were used to compare with RDT results.

#### Therapy

After samples were obtained, empirical therapy was administered immediately according to the empirical diagnosis. The therapeutic regimen was adjusted according to the test result, based on a comprehensive analysis of RDTs and mNGS.

#### Groups

A final diagnosis based on the comprehensive analysis results of all the tests and the effect of the therapy was made. All 58 patients were categorized into two groups defined as infectious uveitis (IU) and non-infectious uveitis (NIU) according to the final diagnosis.

### mNGS of intraocular aqueous samples

Samples were separately collected and sent to the BGI-Wuhan Medical Laboratory (BGI-Wuhan, China) for pathogen mNGS. A 1.5 ml microcentrifuge tube with 100 µl sample and 250 µl of 0.5 mm glass beads was attached to a horizontal platform on a vortex mixer and agitated vigorously at 2800–3200 r.p.m. for 30 min. Then, 7.2 µl lysozyme was added for the wall-breaking reaction. A 300 µl sample was separated into a new 1.5 ml microcentrifuge tube and DNA was extracted using the TIANamp Micro DNA Kit (DP316, Tiangen Biotech) according to the manufacturer’s recommendation.

The extracted DNA was first fragmented to yield 150 bp fragments with enzymatic digestion (RM0434, BGI Wuhan Biotechnology). For construction of the DNA library, fragmented DNA was further end-repaired, ligated to adapters and amplified using PCR with the PMseq high-throughput gene detection kit for infectious pathogens (combined probe anchored polymerization sequencing method, RM0438, BGI-Shenzhen), according to the manufacturer’s instructions. Based on the qualified dsDNA library, a single-stranded circular DNA library was then generated through DNA denaturation and circularization. DNA nanoballs (DNBs) were then formed by rolling circle amplification (RCA) using a universal kit for sequencing reaction (RM0170, Combinatorial Probe-Anchor Synthesis, BGI-Shenzhen). DNBs were qualified by a Qubit ssDNA Assay Kit (Thermo Fisher Scientific) and were further sequenced by the MGISEQ-2000 platform (MGI). For each sequencing run, NTC (no template control), PC (positive control), and both RNA and DNA libraries were processed in parallel. Steps included nucleic acid extraction, library construction, DNB formation and rapid-run sequencing on an MGIseq 2000 instrument, targeting 15–20 million sequences per library.

High-quality sequencing data were generated by eliminating low-quality reads, adapter contamination and short reads (<35 bp), ensuring a minimum of 20 million sequencing reads per library. Human host sequences were computationally subtracted by mapping them to the human reference genome (hg19) using Burrows-Wheeler Alignment [13]. The remaining data were then classified by aligning simultaneously to the Pathogens Metagenomics Database (PMDB), consisting of bacteria, fungi, viruses and parasites. The classification reference databases were downloaded from NCBI (ftp://ftp.ncbi.nlm.nih.gov/genomes/); they contained 4945 whole genome sequences of viral taxa, 6350 bacterial genomes or scaffolds, 1064 fungi related to human infection and 234 parasites associated with human diseases.

All statistical analyses were performed using SPSS 25.

### Criteria for positive results of mNGS

The number of reads refers to the number of strictly aligned sequences detected at the genus/species level. All organisms identified in one sample must be sorted according to the reads mapping to each organism. The top ten list was obtained for further analysis. For bacterial (mycobacteria excluded) and fungal cases, the mNGS result was considered positive when the number of reads mapping to a micro-organism (species level) was ≥3. In contrast, a positive mNGS was given to virus cases if the number of reads aligning to a micro-organism (species level) was ≥1. For parasites, mNGS results were considered positive when the number of reads reached 100. Because the content of *Mycobacterium tuberculosis* complex (MTBC) in blood samples was very low, and the possibility of environmental pollution was extremely low, the result could be considered positive as long as mNGS detected at least one unique read from MTBC.

## Results

### Sample and patient characteristics

Features of the sample in the current study are provided in Table 1. We recruited 58 cases suspected of uveitis based on our inclusion/exclusion criteria. All these cases were categorized into two groups defined as IU (32 cases) and NIU (26 cases) according to the final diagnosis. We took 25 aqueous humour samples and seven vitreous humour samples in IU group patients, and 24 aqueous humour samples and two vitreous samples in the NIU group. The detail information for all cases was shown in Table S1, available in the on line version of this article

**Table 1.** Demographic features of the patients

		IU	NIU
Sample	Aqueous humour	25	24
	Vitreous humour	7	2
Categories	Retinitis	16	12
	Endophthalmitis	11	0
	Choroiditis	2	4
	Posner–Schlossman syndrome	2	2
	Panuveitis	0	4
	Anterior uveitis	1	3
	Vitreous haemorrhage	0	1

According to the clinical manifestations, we categorized all the cases into seven categories, retinitis (16 IU cases, 12 NIU cases), endogenous/exogenous endophthalmitis (11 IU cases, no NIU case), choroiditis (2 IU cases, 4 NIU cases), Posner–Schlossman syndrome (2 IU cases, 2 NIU cases), panuveitis (no IU case, 4 NIU cases), anterior uveitis (1 IU case, 3 NIU cases cases) and vitreous haemorrhage (no IU case, 1 NIU case).

### Diagnostic performance comparison of mNGS and RDTs

#### Comparison of diagnostic performance for differentiating IU from NIU

**Fig. 1.**
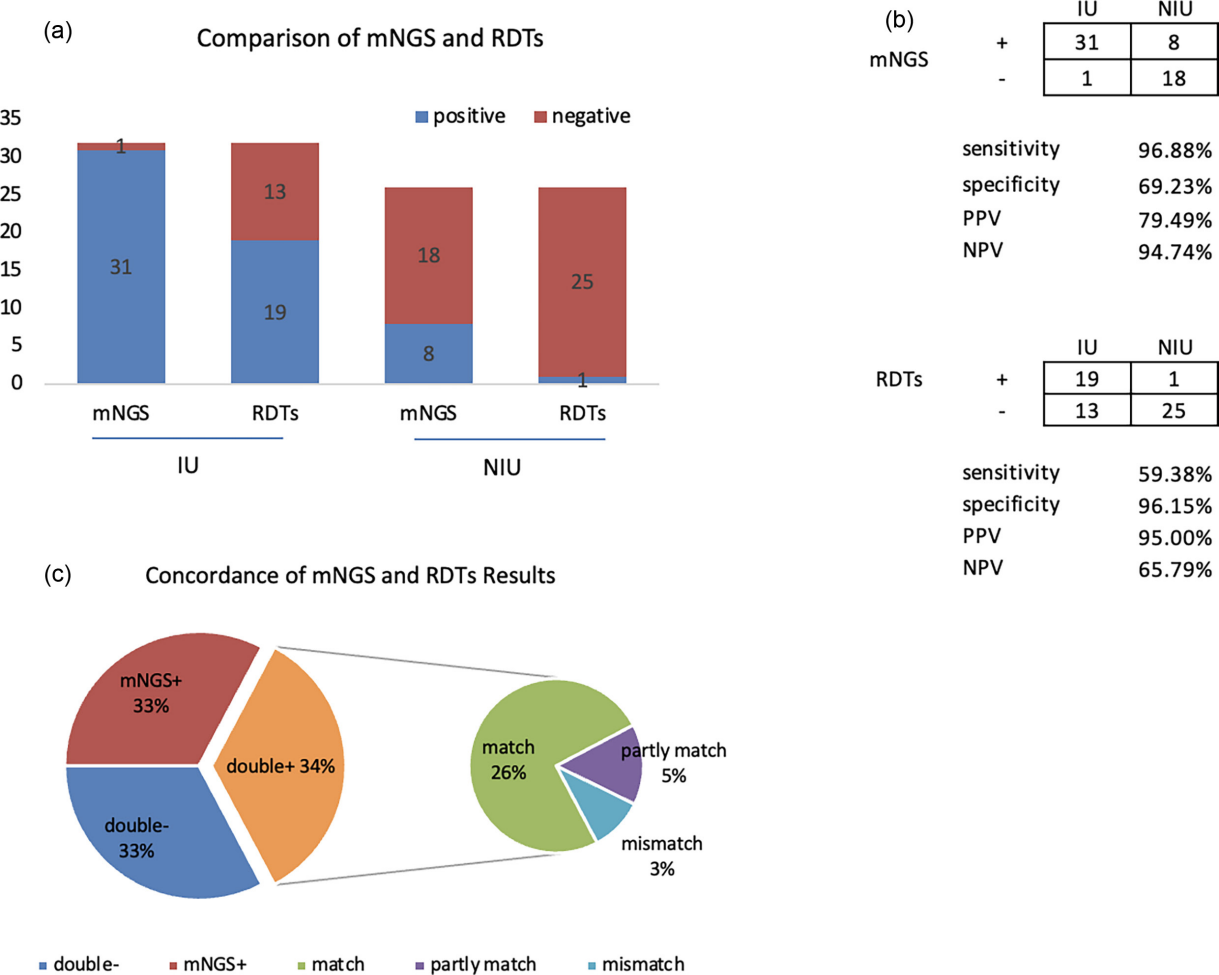
Diagnostic performance comparison of mNGS and RDTs. (a, b) Comparison of the sensitivity and specificity of mNGS and RDTs. The sensitivity and specificity of mNGS were 96.88 and 69.23%, while the rates of RDTs were 59.38 and 96.15%. (c) Concordance between mNGS and RDTs for pathogen detection. mNGS and RDTs were both positive in 34% of cases and both negative in 33% of cases. In 33% of cases, mNGS was the only method that was positive.

Positivity and negativity rates of mNGS and RDTs for IU and NIU are illustrated in Fig. 1(a, b). Fifty-eight samples were utilized to compare the diagnostic efficiency in differentiating IU from NIU. In contrast to RDTs, mNGS increased sensitivity from 59.38 to 96.88% while decreasing specificity from 96.15 to 69.23%. The negative predictive values and positive predictive values of diagnosing infectious uveitis by mNGS were 94.74 and 79.49%, respectively.

#### Concordance between mNGS and RDTs for pathogen detection

**Fig. 2.**
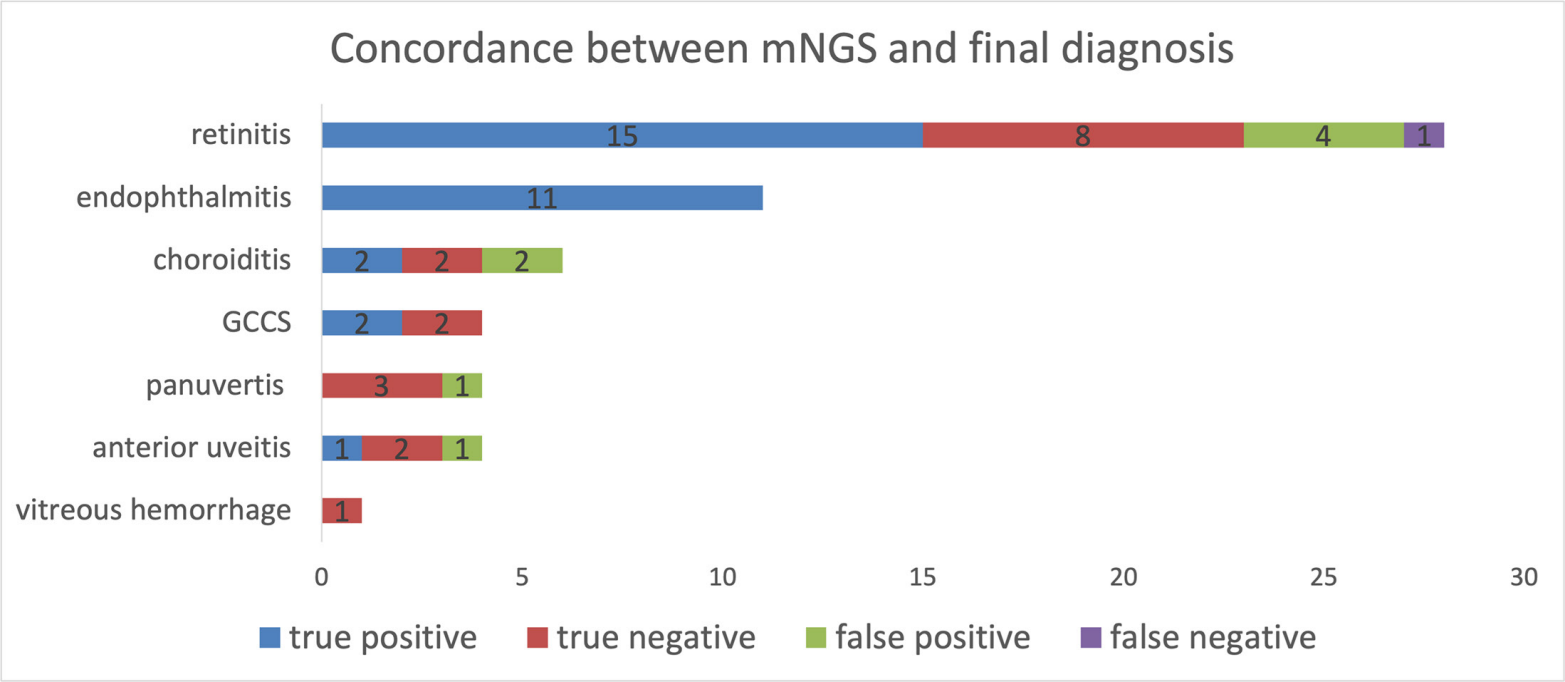
The concordance between mNGS and final diagnosis. The matching rate of mNGS in diagnosing retinitis, endogenous/exogenous endophthalmitis, choroiditis, Posner–Schlossman syndrome, panuveitis, anterior uveitis and vitreous haemorrhage was 82.14, 100, 66.67, 100, 75, 75 and 100%, respectively.

mNGS and RDTs were both positive in 20 of 58 (34%) cases and were both negative in 19 of 58 (33%) cases. Nineteen samples were positive by mNGS only (33%), but none were positive by RDTs only (0%). For double-positive samples (20 cases in total), the two results were completely matched in 15 cases; three cases were found to be ‘partly matched’, indicating at least one overlap of pathogens when polymicrobial results were observed. The remaining two cases were completely mismatched (Fig. 2c). Details of these two cases were as follows.

Case 1 was a patient who was initially suspected of having viral retinitis or endophthalmitis. mNGS, culture and PCR for herpes simplex virus (HSV) and varicella zoster virus (VZV) were performed. mNGS of aqueous humour showed a positive result for Epstein Barr virus (EBV) and cytomegalovirus (CMV), while culture of the aqueous humour showed a positive result for *K. pneumoniae* and the PCR for HSV and VZV was negative. These results appeared to be contradictory. To further verify the accuracy of the results, PCR for EBV and CMV and culture again were performed. PCR for CMV was positive, but EBV was negative, and culture on this occasion was negative. Combined with the patient’s history of renal transplantation, and effective antiviral treatment (without using antibiotics), the final clinical diagnosis was viral retinitis, infective uveitis. The mNGS positive was a true positive, but the RDT positive (culture positive for *K. pneumoniae*) was a false positive, indicating a double-positive mismatch.

Case 2 was a patient who was initially suspected to have retinal vasculitis or infection of the retina. Thus, mNGS, T-spot, PPD and a CT scan of the lung were performed. mNGS test results were positive for *Bremia lactucae*, *Aspergillus sydowii* and *Candida parapsilosis*, but the RDTs of T-SPOT, PPD and CT were all positive, indicating an extraocular tuberculosis infection. Combined with the effective anti-tuberculosis treatment, the final clinical diagnosis was tuberculous vasculitis, NIU. The mNGS positive was a false positive, but the RDT positive (T-SPOT, PPD and CT) was a true positive, indicating a double-positive mismatch.

### Concordance between mNGS and final diagnosis

According to the final diagnosis and its involved lesion due to the type of uveitis, all the final diagnoses were categorized into seven groups defined as anterior uveitis (4 cases), vitreous haemorrhage (1 case), panuveitis (4 cases), endogenous/exogenous endophthalmitis (11 cases), retinitis (28 cases), choroiditis (6 cases) and Posner–Schlossman syndrome (4 cases). For the 28 retinitis cases, the mNGS results of 23 cases (82.14%) matched well with the diagnosis. The matching ratios were 100, 66.67, 100, 75, 75 and 100% for endogenous/exogenous endophthalmitis, choroiditis, Posner–Schlossman syndrome, panuveitis, anterior uveitis and vitreous haemorrhage, respectively. When we divided the cases into NIU and IU groups according to the final diagnosis, the matching ratio was 69.23% (18/26) and 96.88% (31/32), respectively.

We recruited 28 retinitis cases in total, 23 of which matched the final diagnosis. In these 23 cases, 18 were diagnosed with IU and received true positive mNGS results while the other five were diagnosed with NIU and received true negative mNGS results. However, there were five cases in which mNGS results did not match the final diagnosis: four cases were diagnosed with IU but received negative mNGS results, indicating a false negative, and one case was diagnosed with NIU but received a false positive. This concordance between mNGS and the final diagnosis is shown in Fig. 2.

### False positive/negative cases of mNGS

The false positive and false negative cases of mNGS are detailed in Table 2. There were eight false positive cases, which were finally diagnosed with NIU. *Candida* was the most common false pathogen detected by mNGS which appeared in our cases (case Nos. 32, 35, 41 and 42). There was also one false negative case, which involved a patient with posterior focal retinitis who initially had a negative mNGS result using aqueous humour. This patient ultimately underwent vitrectomy, but showed a positive mNGS result as an infection with *Staphylococcus epidermidis* using vitreous humour.

**Table 2.** False positive/negative cases

Patient no.	Sample type	Clinical diagnosis	mNGS result
7	Aqueous humour	Retinitis	Human herpes virus 7
12	Aqueous humour	Retinitis	None
17	Aqueous humour	Anterior uveitis	*Cellomonas*
19	Aqueous humour	Panuvertis	JC polyomavirus, *Streptococcus pneumoniae*, *Nocardia cuticola*
32	Aqueous humour	Choroiditis	*Candida*
35	Aqueous humour	Choroiditis	*Acinetobacter baumannii*, *Staphylococcus aureus*, *Pseudomonas aeruginosa*, *Escherichia coli*, *Aspergillus*, *Candida*
41	Vitreous humour	Retinitis	*Bremia lactucae*, *Aspergillus*, *Candida*
42	Aqueous humour	Retinitis	*Candida*
51	Aqueous humour	Retinitis	*Pseudomonas aeruginosa*

### Comparative analysis of mNGS and RDTs for pathogens

**Fig. 3.**
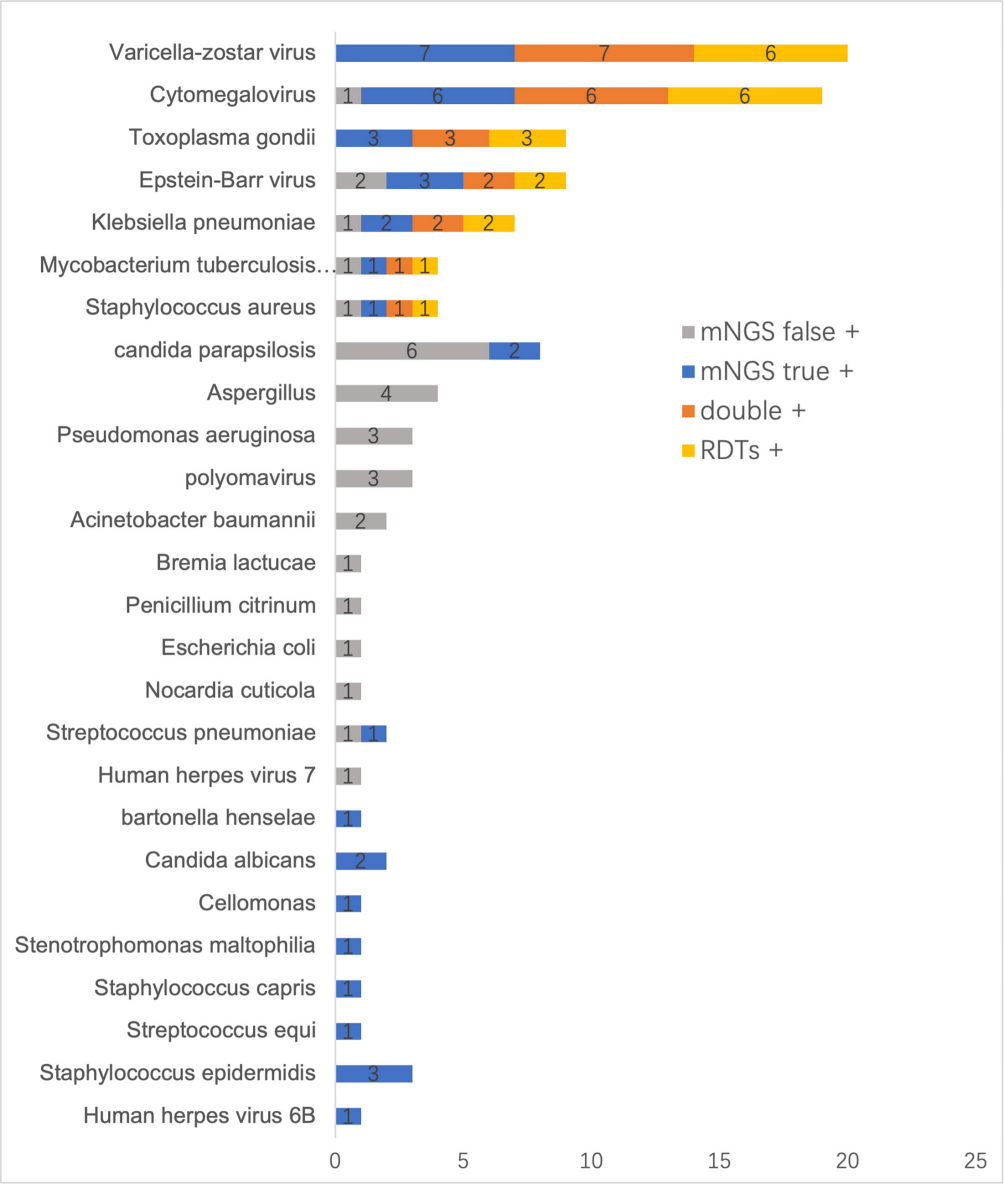
Comparison of mNGS and RDTs by pathogens. Pathogens that could be detected by mNGS are labelled as mNGS+ (according to the final diagnosis, mNGS+ could be categorized as mNGS true+ or mNGS false+). Pathogens that could be detected by RDTs are labelled as RDTs+. Pathogens that could be detected by both mNGS and MDTs are labelled as double+.

For a single sample, mNGS usually can test several potential pathogens rather than a particular one. We assessed every pathogen that was detected by mNGS, even if it was not the final confirmed causative pathogen, containing true positives and false positives, and compared this with RDTs. The pathogens that could be detected by mNGS were labelled as mNGS+ (according to the final diagnosis, mNGS+ could be categorized as mNGS true+ or mNGS false+). The pathogens that could be detected by RDTs were labelled as RDTs+. The pathogens that could be detected by both mNGS and MDTs were labelled as double+. The result is shown in Fig. 3. We detected 26 types of pathogens in total, including viruses, bacteria, fungi and parasites.

## Discussion

Identifying the pathogenesis of uveitis is crucial for treatment and prognosis. The existing paradigm using RDTs for distinguishing between infections and non-infectious uveitis is particularly challenging due to the limited availability and volume of intraocular fluid samples. In this prospective study, we demonstrate the microbiological diagnostic performance of mNGS technology in detecting the IU pathogen.

mNGS had a sensitivity of 96.88% despite requiring an extremely small volume of sample. Only one case of the 32 IU cases was missed using mNGS. This involved a patient with posterior focal retinitis who initially had a negative mNGS result using aqueous humour, then underwent vitrectomy, but showed a positive mNGS result as a *Staphylococcus epidermidis* infection using vitreous humour. mNGS was effective in detecting not only common micro-organisms, such as common bacteria, fungi and viruses, but also rare pathogens, such as *B. henselae*, *T. gondii* and MTBC. For cases that were highly suspected to be IU, such as exogenous or endogenous endophthalmitis, focal retinitis, acute retinal necrosis, granulomatous choroiditis and Posner–Schlossman syndrome, mNGS results were highly concordant with the clinical manifestations. Due to the limited volume of intraocular fluid samples, however, the detection rate with RDTs was quite low. For IU, mNGS is vital to elucidate the species of pathogenic micro-organism involved to guide the final therapy.

The considerable sensitivity of mNGS depended on its detection principle. Culture has historically been the gold standard for the diagnosis of infectious diseases, especially for certain types of bacterial and fungal infections, but there are limitations to the availability and volume of intraocular fluid samples [9]. With the poor sensitivity, and laborious and time-consuming nature of culture methods, faster, streamlined and more sensitive molecular diagnostic tools have been developed for use in diagnosing IU. *T. gondii*, CMV, HSV and VZV are some of the most common pathogens that cause IU. They can also be used to detect bacteria or fungi using targeted species-specific or broad-range universal PCR assays. Nevertheless, PCR is only useful in cases where there are already strong diagnostic clues to suggest certain causative pathogens so that assays targeting the potential infectious agents can be performed [14]. Due to the diversity of infection types, however, it was difficult for clinicians to design an appropriate RDT method. The potential of mNGS to overcome the limitation of culture and PCR is unprecedented. It can possibly detect any pathogen present in a sample of intraocular fluid even in the tiny volume of fluid.

mNGS caused eight false positive cases mostly due to its low specificity of 69.23%. The false positive cases are shown in Table 2. The low specificity made it intractable for clinicians to determine the veracity of positive mNGS results. Given the ultrahigh sensitivity for all types of DNA, even if there are only a few copies, sampling and the lab environment may be a source of significant interference. Designing controls from the sampling and laboratory environment to eliminate interference could be an effective method for enhancing specificity. Creating an environmental biological database for the purpose of optimizing an artificial intelligence algorithm and comparing it with the clinical sample results may also be an effective way to eliminate environmental interference. Combining the history, clinical presentation, effect of empirical treatment and other RDTs to confirm a positive result is another good choice. Collecting the sample again and designing a PCR for validation based on a positive mNGS result may be more effective in enhancing the accuracy.

mNGS also showed an outstanding negative predictive value of 94.74%, indicating that mNGS negative results are likely to be accurate negatives. In other words, a negative mNGS result is significantly more useful for identifying NIU.

## Conclusion

The detection rate of IU pathogens was significantly enhanced by mNGS, as compared to conventional diagnostic testing. However, its reduced specificity also caused some interference in clinical diagnosis. The excellent negative predictive value suggested that the identification of NIU would be of considerable importance. In the future, it will be necessary to carry out relevant optimization and secondary verification of its detection results to improve the detection efficiency of this detection method.

## supplementary material

10.1099/jmm.0.001879 Uncited Table S1.

## Abbreviations

CMV: cytomegalovirus
CT: computed tomography
DNB: DNA nanoball
EBV: Epstein–Barr virus
HHV: human herpes virus
HSV: herpes simplex virus
IU: infectious uveitis
mNGS: metagenomic next-generation sequencing
MTBC: *Mycobacterium tuberculosis* complex
NIU: non-infectious uveitis
PCR: polymerase chain reaction
PPD: purified protein derivative
RCA: rolling circle amplification
RDT: routine diagnostic test
VZV: varicella zoster virus
